# Three-dimensional reconstruction and intraoperative ultrasonography: Crucial tools to safely approach highly complex renal masses

**DOI:** 10.1590/S1677-5538.IBJU.2022.0224

**Published:** 2022-05-18

**Authors:** Antonio Andrea Grosso, Luca Lambertini, Fabrizio Di Maida, Maria Lucia Gallo, Andrea Mari, Andrea Minervini

**Affiliations:** 1 University of Florence Unit of Oncologic Minimally Invasive Urology and Andrology Department of Experimental and Clinical Medicine Florence Italy Department of Experimental and Clinical Medicine, University of Florence - Unit of Oncologic Minimally Invasive Urology and Andrology, Careggi Hospital, Florence, Italy

## Abstract

**Purpose:**

Robot-assisted partial nephrectomy (RAPN) is rapidly increasing its role in the nephron-sparing surgery setting (1). The recent introduction of technological advancements is leading more experienced surgeons to approach complex renal mass with a conservative intent (2, 3).

In particular, three-dimensional reconstruction and the use of intraoperative ultrasonography are gaining attention as crucial tools to safely and effectively approach complex cases (4, 5).

We aimed to video-report the management of highly complex renal mass treated with RAPN, focusing on preoperative surgical planning and intraoperative technical nuances.

**Materials and methods:**

A 73-year-old male patient was referred to our institution for an incidental detection of a 70 mm diameter, completely endophytic, hilar renal mass (PADUA score 13, RENAL score 11a). Contrast-enhanced CT scan images were processed by M3DICS (Turin, Italy) and used to obtain a 3D virtual model. RAPN was performed by a highly experienced surgeon using Da Vinci Si robotic platform with a three-arm configuration.

**Results:**

The overall operative time was 114 min, with a warm ischemia time of 16 min. No intraoperative or postoperative complications were recorded. According to the SIB score, the pure enucleation excision strategy was performed. Histopathological analysis revealed a pT3a low-grade oncocytic kidney tumor with negative surgical margins. with negative surgical margins. At 24-months follow up, no local or systemic recurrence was detected.

**Conclusions:**

Conservative management of complex renal masses is challenging with a highly nuanced decision-making process. In this regard, preoperative 3D models and intraoperative ultrasound (US) guidance play a pivotal role to develop a tailored surgical strategy according to patient’ and tumor's characteristics.

## INFORMED CONSENT

Informed consent was obtained from all individual participants included in the study. All the procedures were in accordance with the ethical standards of the institutional and national research Committee and with the 1964 Helsinki declaration and its later amendments or comparable ethical standards.
